# Association of Bullous Lichen Sclerosus and Morphea

**DOI:** 10.1002/ccr3.70315

**Published:** 2025-03-17

**Authors:** Seyyede Zeinab Azimi, Fatemeh Sari Aslani, Mohammad Mahdi Parvizi, Mohammad Reza Namazi

**Affiliations:** ^1^ Center for Research & Training in Skin Diseases & Leprosy Tehran University of Medical Sciences Tehran Iran; ^2^ Molcular Dermatology Research Center, Department of Pathology School of Medicine, Shiraz University of Medical Sciences Shiraz Iran; ^3^ Molecular Dermatology Research Center, Department of Dermatology, Shiraz University of Medical Sciences Shiraz Iran; ^4^ Dr. Namazi Skin and Hair Clinic Shiraz Iran

**Keywords:** breast cancer, bullous, lichen sclerosus, morphea

## Abstract

Lichen sclerosus (LSc) is an inflammatory skin disease of unknown etiology. The coexistence of LSc and morphea in the same lesion is uncommon but exists. Also, there exist a few rare cases of bullous LSc–generalized morphea overlap syndrome.

## 1 Introduction

Lichen sclerosus (LSc) and morphea are chronic inflammatory cutaneous diseases with unknown etiology and pathogenesis. LSc usually presents as white, porcelain‐like, atrophic plaques in the anogenital area of postmenopausal women. Extragenital lesions may sometimes be present. Bullous types of both morphea and LSc are rarely reported [1, 2, 3, 4]. Herein, we presented a rare manifestation of LSc (bullous LSc) on generalized morphea plaques in a woman with a previous history of malignancy.

## 2 Case History

**FIGURE 1:**
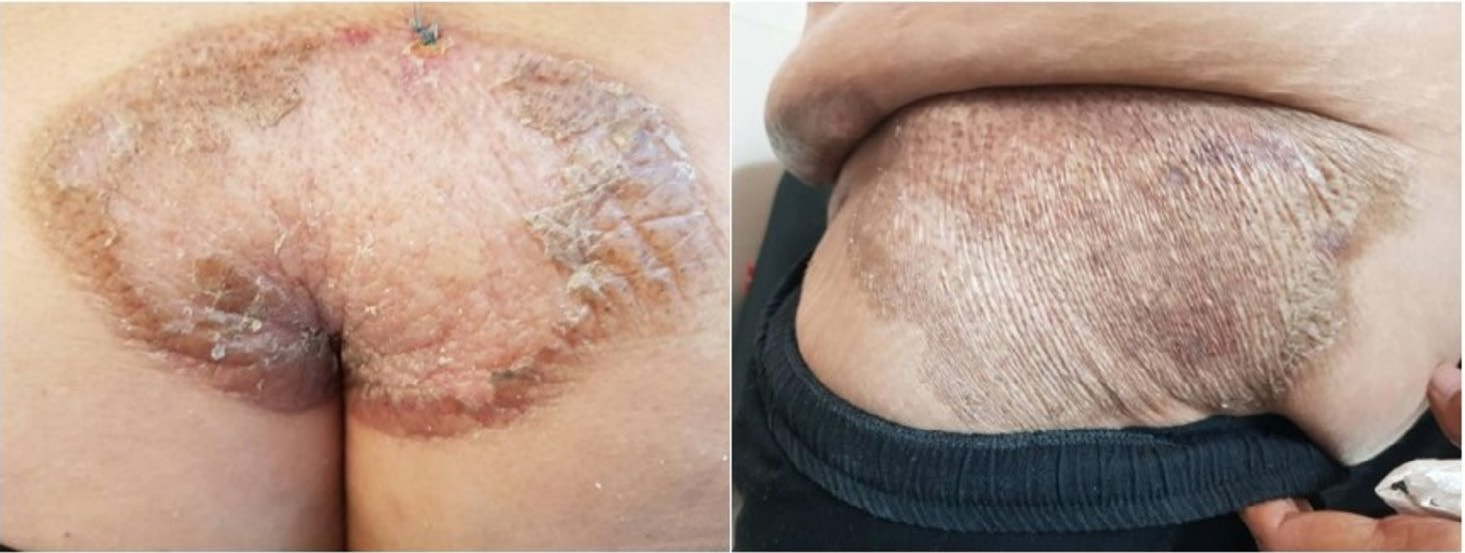
Large, intensely indurated plaques with white areas on the buttock (A) and abdomen (B).

**FIGURE 2:**
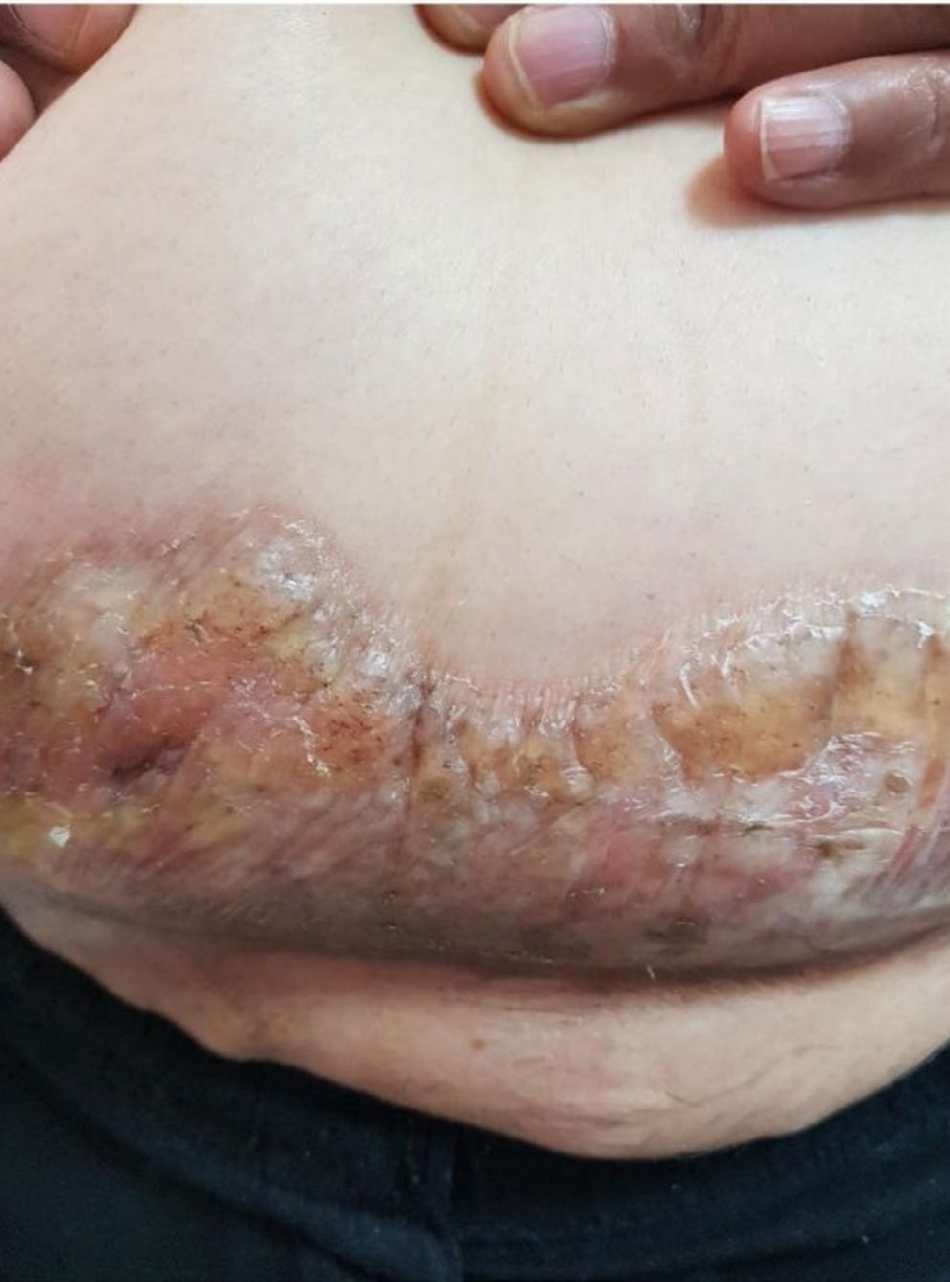
Bullous lesions on the patient's morphea plaques.

A 65‐year‐old woman with a history of breast cancer treated by radical mastectomy 8 years earlier was referred to our department for progressive formation of bullous lesions on the indurated skin. She did not received radiotherapy after her mastectomy and was taking no drugs. A few months after the diagnosis of her breast cancer, she developed generalized plaque morphea (Figure 1). She had no pruritus on the morphea plaques. From 4 months ago, she developed bulla lesions on the morphea plaques of the lower abdomen (Figure 2). These lesions were painful. Clinical examination showed tender erythematous indurated plaques containing bullae on the lower abdominal area, in addition to the nonbullous erythematous and sclerotic plaques on the posterior trunk and buttock.

## 3 Methods

**FIGURE 3:**
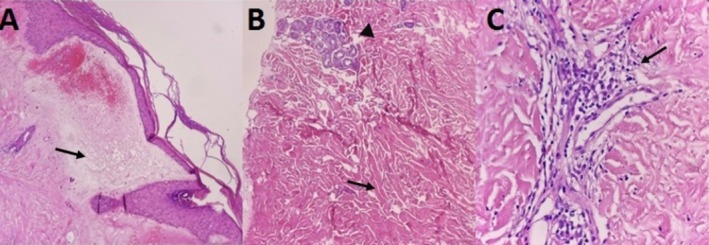
There is hyperkeratosis, thinning of the epidermis, severe papillary dermal edema (arrow), hemorrhage, subepidermal bulla, homogenization of collagen fibers, and prominent thin‐walled vessels (A). The reticular dermis shows thickening of collagen bundles (arrow) with perieccrine fat replacement (arrow head) (B) and mild perivascular lymphocyte and plasma cell infiltration (arrow) (C). (H&E ×100 & 400).

The biochemical laboratory tests and chest x‐ray were not noticeable. An incisional biopsy was taken from the bullous abdominal lesion. A consent form was obtained from the patient. Microscopic examination of the trunk skin lesion showed hyperkeratosis, thinning of the epidermis, severe edema of the dermal papillae, hemorrhage, subepidermal bulla, homogenization of collagen fibers, and prominent thin‐walled vessels. There was mid‐dermal mild perivascular and focal perifollicular lymphocyte and plasma cell infiltration as well as thickening of collagen bundles of the reticular dermis (Figure 3).

## 4 Conclusion

According to the mentioned histopathological findings, simultaneous features of both bullous LSc and morphea were seen in the same lesion.

## 5 Discussion

The prevalence of LSc is 0.1% to 0.3%, bullous LSc is a rare variant of LSc, and its epidemiology has not been reported [5]. The incidence of morphea differs between 0.34 and 2.7 cases per 100,000 population annually [6]. Genetic susceptibilities, trauma, infections such as human papilloma virus and spirochaetes, autoimmune mechanisms, and rarely the administration of vaccines have been suggested as causative factors [2].

Some cases of coexistence of morphea and LSc have been reported before [7, 8, 9, 10, 11, 12, 13, 14, 15, 16]. However, the word coexistence continues to be controversial, as some authors think LSc is a type of morphea, with prominent superficial involvement, while others categorize the diseases both clinically and histopathologically as two distinct diseases [10, 14, 15].

In LSc, prominent findings are edema, paling, and collagen homogenization in the papillary dermis; whereas in morphea, the reticular dermis is also affected, and the parallel arrangement of coarse collagen bundles beside the atrophy in skin appendages is also obvious [17].

Yasar et al. reported a 70‐year‐old patient with annular atrophic plaques on both sides of the trunk that were ivory colored in the middle and surrounded by erythema. The plaques occasionally developed bulla, and the histopathologic examination of bullous lesions revealed bullous morphea and LSc [2]. Sadati et al. published a 67‐year‐old case of generalized morphea with some plaques containing bullae on the anterior trunk and inguinal area, which microscopic examination showed concomitant features of both bullous LSc and morphea in the same lesion [18]. Liu et al. reported a case of a 50‐year‐old man with bullous LSc complicated later with generalized morphea [19].

Clinically, bullous morphea may be similar to bullous LSc, and the clinical setting is inadequate for the definitive diagnosis. The diagnosis will be made by histological examination in which only bullous LSc shows hyperkeratosis, follicular plugging, or epidermal atrophy with vacuolar change of basal cells [18], as seen in our case.

The coexistence of morphea and bullous LSc is a rare finding, but clinicians should draw attention to this uncommon association. Also, this association strengthens the possible etiological link between them.

## Author Contributions

**Seyyede Zeinab Azimi:** data curation, supervision, writing – original draft, writing – review and editing. **Fatemeh Sari Aslani:** data curation, investigation, supervision, writing – review and editing. **Mohammad Mahdi Parvizi:** investigation, writing – review and editing. **Mohammad Reza Namazi:** conceptualization, data curation, investigation, supervision, writing – review and editing.

## Consent

Written informed consent has been taken from the patient.

## Conflicts of Interest

The authors declare no conflicts of interest.

## Data Availability

Available as needed.
